# Supplementary material for: Causal Relationship between Aging and Anorexia Nervosa: A White-Matter-Microstructure-Mediated Mendelian Randomization Analysis
Source: Biomedicines. 2024 Aug 16;12(8):1874. doi: 10.3390/biomedicines12081874 (PMC11351342; doi:10.3390/biomedicines12081874)
Supplement: Supplementary file 1 [file biomedicines-12-01874-s001.zip › Supplementary Figure/Figure S4/Singe SNP plot of MR analysis for TL to ubm-b-1704.pdf]

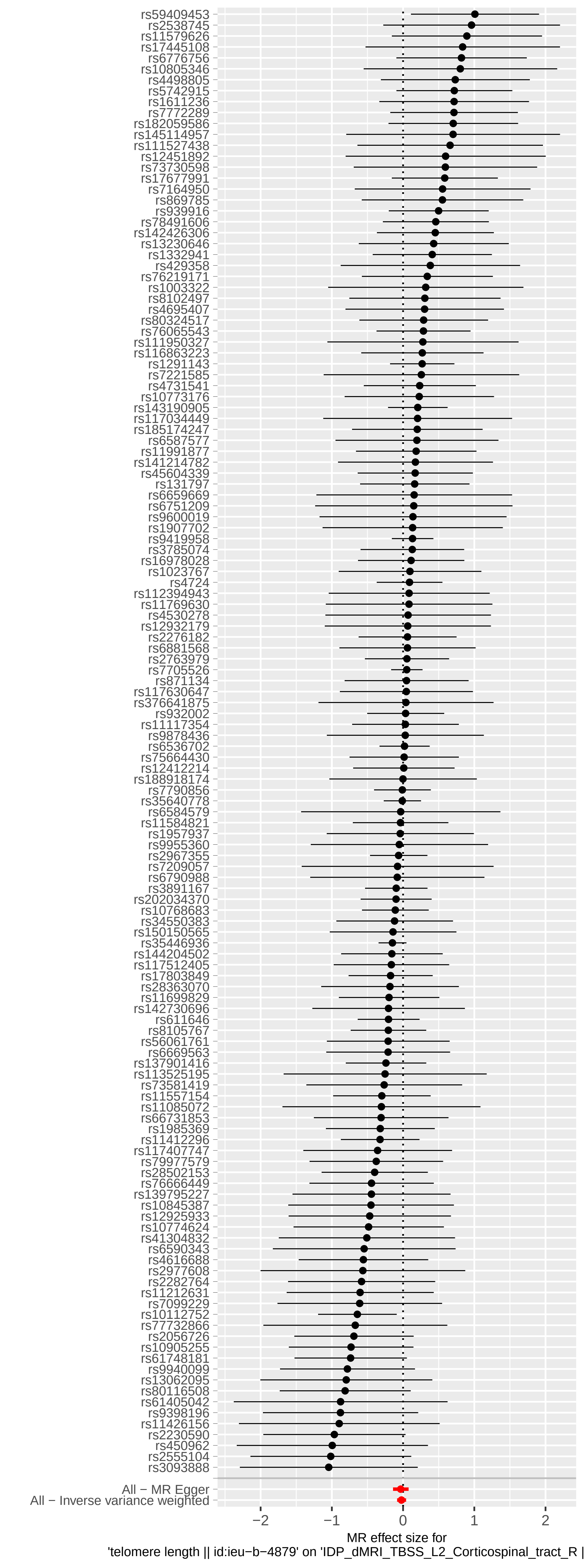
rs59409453
rs2538745
rs11579626
rs17445108
rs6776756
rs10805346
rs4498805
rs5742915
rs1611236
rs7772289
rs182059586
rs145114957
rs111527438
rs12451892
rs73730598
rs17677991
rs7164950
rs869785
rs939916
rs78491606
rs142426306
rs13230646
rs1332941
rs429358
rs76219171
rs1003322
rs8102497
rs4695407
rs80324517
rs76065543
rs111950327
rs116863223
rs1291143
rs7221585
rs4731541
rs10773176
rs143190905
rs117034449
rs185174247
rs6587577
rs11991877
rs141214782
rs45604339
rs131797
rs6659669
rs6751209
rs9600019
rs1907702
rs9419958
rs3785074
rs16978028
rs1023767
rs4724
rs112394943
rs11769630
rs4530278
rs12932179
rs2276182
rs6881568
rs2763979
rs7705526
rs871134
rs117630647
rs376641875
rs932002
rs11117354
rs9878436
rs6536702
rs75664430
rs12412214
rs188918174
rs7790856
rs35640778
rs6584579
rs11584821
rs1957937
rs9955360
rs2967355
rs7209057
rs6790988
rs3891167
rs202034370
rs10768683
rs34550383
rs150150565
rs35446936
rs144204502
rs117512405
rs17803849
rs28363070
rs11699829
rs142730696
rs611646
rs8105767
rs56061761
rs6669563
rs137901416
rs113525195
rs73581419
rs11557154
rs11085072
rs66731853
rs1985369
rs11412296
rs117407747
rs79977579
rs28502153
rs76666449
rs139795227
rs10845387
rs12925933
rs10774624
rs41304832
rs6590343
rs4616688
rs2977608
rs2282764
rs11212631
rs7099229
rs10112752
rs77732866
rs2056726
rs10905255
rs61748181
rs9940099
rs13062095
rs80116508
rs61405042
rs9398196
rs11426156
rs2230590
rs450962
rs2555104
rs3093888
All − MR Egger
All − Inverse variance weighted
−2
−1
0
1
2
MR effect size for
'telomere length || id:ieu−b−4879' on 'IDP_dMRI_TBSS_L2_Corticospinal_tract_R |
